# The window of opportunity: a relevant concept for axial spondyloarthritis

**DOI:** 10.1186/ar4561

**Published:** 2014-05-12

**Authors:** Philip C Robinson, Matthew A Brown

**Affiliations:** 1 University of Queensland Diamantina Institute, Translational Research Institute, Princess Alexandra Hospital, Ipswich Road, Woolloongabba 4102, Australia

## Abstract

The window of opportunity is a concept critical to rheumatoid arthritis treatment. Early treatment changes the outcome of rheumatoid arthritis treatment, in that response rates are higher with earlier disease-modifying anti-rheumatic drug treatment and damage is substantially reduced. Axial spondyloarthritis is an inflammatory axial disease encompassing both nonradiographic axial spondyloarthritis and established ankylosing spondylitis. In axial spondyloarthritis, studies of magnetic resonance imaging as well as tumor necrosis factor inhibitor treatment and withdrawal studies all suggest that early effective suppression of inflammation has the potential to reduce radiographic damage. This potential would suggest that the concept of a window of opportunity is relevant not only to rheumatoid arthritis but also to axial spondyloarthritis. The challenge now remains to identify high-risk patients early and to commence treatment without delay. Developments in risk stratification include new classification criteria, identification of clinical risk factors, biomarkers, genetic associations, potential antibody associations and an ankylosing spondylitis-specific microbiome signature. Further research needs to focus on the evidence for early intervention and the early identification of high-risk individuals.

## 

Axial spondyloarthritis (axSpA) is an inflammatory disease of the axial skeleton and pelvis. Regardless of whether it progresses onto ankylosing spondylitis (AS), axSpA has an appreciable disease burden. axSpA is also associated with co-morbidities such as uveitis, psoriasis, inflammatory bowel disease, cardiovascular disease, osteoporosis and significant loss of work productivity. There is emerging evidence that early treatment may change the outcome in axSpA.

The window of opportunity is a concept of critical importance in rheumatoid arthritis (RA). Early treatment results in reductions of disease activity, joint erosions, and better treatment responses the earlier disease-modifying anti-rheumatic drugs are commenced. It also results in a greater proportion of patients in drug-free remission after treatment withdrawal. These findings have led to changes in RA treatment pdigms, with increasing emphasis on early diagnosis and treatment.

So how is this concept relevant to axSpA? A number of studies have demonstrated early treatment that suppresses inflammation may change the outcome of axSpA.

Whilst initial studies suggested that radiographic progression of AS is not slowed by treatment with tumor necrosis factor inhibitor (TNFi) medications, two observational studies have now shown a reduction in radiographic progression with these agents [1,2]. One of these studies also showed that delay in starting TNFi medications was associated with greater radiographic progression [2]. Magnetic resonance imaging (MRI) studies have also supported the link between inflammation and progression of ankylosis. Acute inflammatory lesions are more likely to progress to chronic fatty lesions than areas devoid of inflammation [3]. Vertebral corners with inflammation on MRI are more likely to progress to syndesmophytes than those without [4]. There is good evidence that all of the TNFi medications reduce MRI-detected inflammatory lesions.

Intriguingly there is also new evidence that the age of the inflammatory lesion may influence progression to ankylosis, suggesting that a longer duration of inflammation is associated with more syndesmophyte formation. Maksymowych and colleagues have shown that early, acute type A lesions, without fatty metaplasia, infiltration or erosion, are less likely to progress to syndesmophytes as compared with type B lesions characterized by loss of signal at the vertebral corner [5]. This loss of vertebral corner signal is postulated to be erosion, sclerosis, reption or fatty infiltration, and may be a sign of a more longstanding mature inflammatory lesion. This work supports the theory that early corner lesions in the spine which have not developed reptive changes of fat infiltration can potentially regress, whilst more advanced corner lesions with signs of fatty reptive change are more likely to progress to ankylosis. This work potentially marks fat metaplasia as an event that precedes ankylosis and indicates that suppression of fatty change by treatment may slow progression to ankylosis.

Taken together, these observations provide strong circumstantial evidence that treatment, especially early effective treatment, may influence radiographic outcome.

Trials of TNFi therapy in early axSpA have yielded encouraging results, with greater treatment responses than in disease of longer duration. In Barkham and colleagues’ study of very early axSpA (mean symptom duration 15.3 months), infliximab achieved an Assessment of SpondyloArthritis international Society (ASAS) partial remission rate of 56% [6], compared with 22% in the registration trial of infliximab in established AS (the ASSERT study). Similarly, in the INFAST study of infliximab treatment in axSpA patients with disease duration less than 3 years (mean 1.8 to 1.9 years), higher numerical response rates (40% improvement from baseline using the ASAS criteria (ASAS40) of 75%) were observed than in the ASSERT study (ASAS40 of 45%) or trials of the other TNFi agents in established AS (ASAS40 responses of 39 to 47%) [7-10]. In the ESTHER trial of etanercept, where symptom duration was a mean 2.9 years, ASAS40 was achieved in 70% of patients, compared with ASAS40 of 45% for the etanercept trial in established AS [8,11]. Although one should note that these efficacy comparisons are not head to head in the same trials, they are consistent with a difference in response based on disease duration.

Withdrawal studies of TNFi agents in early axSpA and AS also suggest that the frequency of drug-free remission is higher the earlier a patient is treated. In established AS patients treated with a TNFi agent and monitored for 6 months or more after discontinuation, 91 to 100% of patients flare [12-14]. In the INFAST trial, 40 to 48% of patients who received early infliximab treatment remained in drug-free remission 6 months after stopping their infliximab [15]. In the Barkham and colleagues study mentioned above of infliximab in early axSpA, after only four infusions 33% (four of 12) of patients remained symptom free 5 years after treatment, whereas 100% (13 of 13) of patients initially randomized to placebo were still requiring TNFi therapy [6,16]. The definition of flare varies between studies, and standardization of this definition as well as other issues relating to trial design would enable better conclusions to be drawn. However, the data do suggest that early treatment may change the prognosis of disease. The difference between the studies may relate to study design or exactly how large the window of opportunity is.

The major challenge now remains to accurately identify patients who have poor prognosis. The ASAS axSpA criteria have gone some way to enabling earlier identification of early axSpA but do have some limitations including the ability to capture self-limiting disease [17,18]. Clinical predictors of radiographic progression identified to date include elevated C-reactive protein, tobacco smoking, and the presence of baseline syndesmophytes. Other potentially promising research findings that may enable better identification of patients who will have persisting or bad prognosis disease include biomarkers such as vascular endothelial growth factor, matrix metalloprotein-3, sclerostin, citrullinated vimentin and dikkopf-1. Antibodies to major histocompatibility complex class II-associated invariant-chain peptide have been associated with axSpA by two independent groups; if their specificity is validated in larger studies and they become commercially available, these antibodies hold great promise.

Emerging work examining the gut microbiome of axSpA patients may provide information not only about etiology, but also about prognosis. A distinct microbial signature has been associated with AS (M Brown, unpublished observation). In very recent work Van Praet and colleagues have also found an association between the degree of bowel inflammation, and MRI-demonstrated bone marrow edema of the sacroiliac joints [19].

Finally, clinical prediction from genetic analysis - whilst essentially dominated by HLA-B27 until recently - has progressed significantly, and there are now many independent AS genetic associations [20]. This analysis may become powerful enough in the future to enable prediction of prognosis and particularly radiographic severity.

There may therefore be a time when genetic analysis, probably combined with other biomarkers or clinical features, enables accurate prognostication of disease onset, features and severity. Early, or even potentially prophylactic, treatment could then be applied to stop or attenuate disease.

We should also acknowledge that the data currently available are not conclusive and that a number of studies are required before the evidence becomes compelling. Randomized controlled trials of TNFi therapy withdrawal in very early disease are needed, and some are underway. Longitudinal studies of cohorts of nonradiographic axSpA are required to better understand the natural history of early disease, including remission and progression rates. Further work is required for the development of better biomarkers of early disease and case-identification strategies, which will be required to implement an early intervention strategy.

In conclusion, there is mounting evidence that early effective treatment of inflammation in axSpA can change disease outcome. Identification of high-risk individuals and prompt institution of therapy is going to have increasing importance in clinical practice. We should therefore encourage industry and independent investigators to design and conduct studies in axSpA to confirm the importance of the window of opportunity as well as the accurate identification of high-risk individuals. Early treatment combined with another concept borrowed from RA, treat to target, will hopefully enable patients to enjoy the huge therapeutic progress seen in RA to be translated into axSpA.

## Abbreviations

AS: Ankylosing spondylitis; ASAS: Assessment of SpondyloArthritis international Society; ASAS40: 40% improvement from baseline using the ASAS criteria; axSpA: Axial spondyloarthritis; MRI: Magnetic resonance imaging; RA: Rheumatoid arthritis; TNFi: Tumor necrosis factor inhibitor.

## Competing interests

PCR has received travel support, speaking fees, and research grants, has undertaken clinical trials and has participated in advisory boards for Abbvie, Pfizer, UCB and/or Janssen. MAB has participated in advisory boards for Janssen, Pfizer, Abbvie and UCB, has participated in a speakers’ bureau for Abbvie, and has received research grants and travel support from Abbvie.
